# Health mediators as members of multidisciplinary group practice: lessons learned from a primary health care model programme in Hungary

**DOI:** 10.1186/s12875-020-1092-7

**Published:** 2020-01-28

**Authors:** Karolina Kósa, Cintia Katona, Magor Papp, Gergely Fürjes, János Sándor, Klára Bíró, Róza Ádány

**Affiliations:** 1 Institute of Behavioural Sciences, Faculty of Public Health, University of Debrecen, Móricz Zs. krt. 22, Debrecen, 4032 Hungary; 2 Semmelweis Health Promotion Centre, Budapest, Hungary; 3 Institute of Preventive Medicine, Faculty of Public Health, University of Debrecen, Debrecen, Hungary; 4 Department of Health Management, Faculty of Public Health, University of Debrecen, Debrecen, Hungary

**Keywords:** Prevention, Inequalities, Professions allied to medicine (PAMs), Community health workers, Health mediation, Primary health care, Health status assessment

## Abstract

**Background:**

A Model Programme of primary care group practices was implemented in Hungary between 2013 and 2017 – where virtually all GPs had worked in single practices – aiming to increase preventive service uptake and reduce inequalities based on a bilateral agreement between the Swiss and Hungarian governments. Group practices employed a wide variety of health professionals as well as support workers called health mediators. Employment of the latter was based on two decades of European experience of health mediators who specifically facilitate access to and use of health services in Roma minority groups. Health mediators had been recruited from local communities, received training on the job, and were tasked to increase uptake of new preventive services provided by the group practices by personal contacts in the local minority populace. The paper describes the contribution of the work of health mediators to the uptake of two new services provided by group practices.

**Methods:**

Quantitative analysis of depersonalized administrative data mandatorily reported to the Management of the Programme during 43 months of operation was carried out on the employment of health mediators and their contribution to the uptake of two new preventive services (health status assessment and community health promoting programmes).

**Results:**

80% of all clients registered with the GPs participated at health status assessment by invitation that was 1.3–1.7 times higher than participation at the most successful national screening programmes in the past 15 years. Both the number of mediator work minutes per client and participation rate at health status assessment, as well as total work time of mediators and participants at community health events showed high correlation. Twice as many Roma minority patients were motivated for service use by health mediators compared to all patients. The very high participation rate reflects the wide impact of health mediators who probably reached not only Roma minority, but vulnerable population groups in general.

**Conclusion:**

The future of general practices lays in multidisciplinary teams in which health mediators recruited from the serviced communities can be valuable members, especially in deprived areas.

## Background

### Single-handed vs group GP practices in primary care

General practices may operate as single practices or as groups of self-employed physicians. Group practices comprise the majority in some countries such as Great Britain where 85.5% of all practices were group practices in 2010 [1]. Single-handed practices remain dominant in other countries such as Hungary where all public health insurance-funded practices had been operated single-handedly by GP practitioners up until 2013. The present paper introduces the contribution of a new support worker called” health mediator” of the multidisciplinary GP group practice based on the experiences of a primary health care Model Programme that established group practices in primary care in Hungary since 2013.

### A novel model programme of multidisciplinary group practice in Hungary

Core features of tax-funded primary care in Hungary are similar to that of the UK. Primary medical care is provided by general practitioners [2] who – as opposed to those in the UK – operate single-handed GP practices aided by one or more practice nurses. Coverage is based on a mandatory health insurance scheme with no opting-out [3]. The provision of primary health care is the responsibility of local municipalities, whereas it is financed by the national health insurance fund. The health status of the Hungarian population has long been below the European average [4], partly due to the insufficient uptake of health services in general, and preventive services in particular that has been shown among the largest (Roma) minority [5] as well as in the general population [6].

In order to improve preventive services in primary care, a Model Programme established and funded by the Swiss-Hungarian Cooperation [7] had introduced group practices in primary care in 2012 in the two economically most disadvantaged regions of the country as described in the Operations Manual of the Programme [8] and elsewhere [9]. The Swiss-Hungarian cooperation was set up to promote the reduction of economic and social inequalities within Hungary by implementing mutually agreed projects in various fields. The Model Programme was agreed upon in the field of health care services. Briefly, 24 GP practices were selected on the basis of demographic and population health data, number of vacant GP practices in the region, and willingness of general practitioners and their host municipalities to participate. Four groups of general physicians designated as GP clusters, each consisting of six general practitioners were organized and received funding to employ a range of ancillary health workers such as public health specialists, dietitian, physiotherapist, health psychologist [10]. The specific aims of the Model Programme were to extend public health services in a cost-effective manner to all population groups, particularly disadvantaged ones, to reduce health inequalities. In addition to acute and chronic care, new services were offered (not available in other GP offices) such as lifestyle counselling, nutritional, physiotherapy and psychological services, as well as health promoting community programmes as published in detail elsewhere [11].

An important new service of the group practices was the provision of an invitation-based health status assessment or general screening (investigation of cardiovascular and cancer risk factors – excluding cervical and breast cancer screening organized by a national public health agency –, based on health examination and questionnaire survey) as mandated by law since 1999 for adults on a regular basis depending on age [12] which has not been done systematically by GPs unless specifically requested by their patients/clients. The dismal health status of the Hungarian population, the low uptake of health status assessment available from GPs, the low uptake of preventive services in the general population and in minorities referred to above made it necessary to include health status assessment in the GP clusters’ services at the beginning of the Programme. All adult clients of the GPs were individually invited in writing to the health status assessment carried out by non-medical workers at the beginning of the Programme which was followed by a medical risk assessment carried out by the GP. Based on its results, patients were directed to various paths (further medical examination, or any of the new services: individual or group lifestyle counselling, or specific nutritional, physiotherapy, etc. services, or health promoting community health programmes). Tailored lifestyle counselling and health promoting community programmes were also recommended to patients who had already been in chronic care.

### Employment of support workers called health mediators in primary health care

The involvement of support workers (community workers) with no professional qualification in primary health care has a long history around the world. Chinese peasants with a few months of training (“barefoot doctors”) provided basic care to large segments of the rural population from the 1930s onward. This model was taken up by other developing countries worldwide in order to address the problem of unserved population groups due to shortages of professional health care workers [13]. The World Health Organization recognized the important role of community health workers in providing essential primary health care services and thereby reducing health inequalities, and recently issued policy guidelines for creating relevant programmes [14].

The mostly Roma minority disadvantaged groups of the developed countries of Europe posed a different challenge. Roma minority groups had difficulties accessing and/or utilizing various public services, or/and expressed mistrust in these services. As a means to decrease mistrust and clarify misunderstanding between governmental agencies and minorities, mediation had been introduced in France and Finland in social services in the 1960s with the ultimate aim of increasing the access and uptake of those services [15]. Mediation in primary health care was initiated in Romania during a vaccination project in 1997 when large numbers of Roma refused vaccination due to complex reasons [16]. Since then, health mediators have worked in a number of European health projects, most of them during the Decade of Roma Inclusion [17].

**Fig. 1:**
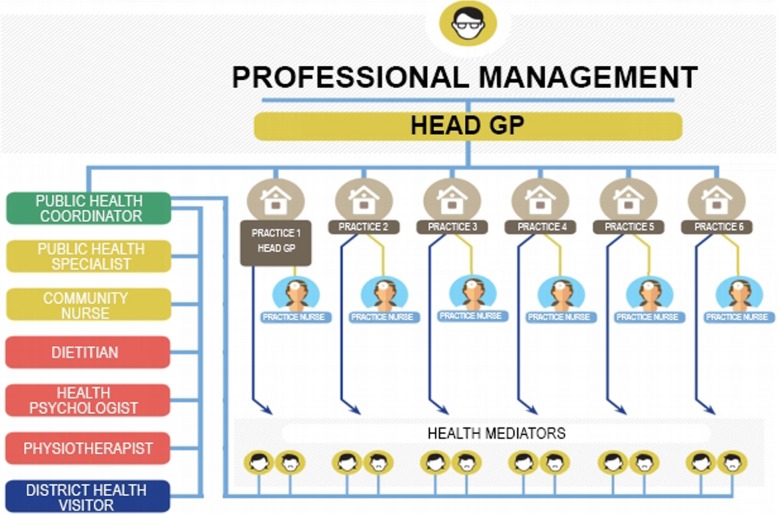
Human resources of group practices (GP clusters)

The inclusion of health mediators in the Model Programme was justified based on two decades of experience with Roma communites [18]. Health mediators were recruited from the local communities and employed part-time with no requirement for professional or vocational training (Fig. 1). They were recruited by public advertisement as prescribed by law and by locally distributed leaflets facilitated by the participating GPs and practice nurses. All health mediators were required to reside in the local community; preference was given to those applicants who identified with or had experience working with the largest minority (Roma) population of the regions. Their major task was to bridge the gap between general practitioners and their socioeconomically vulnerable clients by ensuring individualized support for the latter, counterbalancing the potential increase of health inequalities inherent in the population approach of prevention uncovered by Frohlich and Potvin [19]. The Programme planned to employ 12 mediators per GP cluster or altogether 48 persons (most of them middle-age women living in the local communities and identifying at the time of employment as Roma) on part-time contracts equivalent to 20 work hours per week. Work allocation and supervision of health mediators was the responsibility of the supervisor of all non-medical personnel of the GP cluster, the so-called public health coordinator (a public health professional) who reported to the head of the GP cluster (one of the GPs elected by all GPs from themselves).

### Training for health mediators

Vocational training of 800 h in assistant nursing was provided in the first year of employment to all those health mediators who had the appropriate educational qualification to enter training and who did not yet have any health-related vocational qualification. 3-day training in health mediation was provided to all health mediators in the first and third years of employment in the Programme. All expenses related to both vocational and mediator trainings were fully paid for by the Programme, and both were completed during work hours. A number of short courses of continuing education were also developed for health mediators and completed during work hours.

### Work tasks of health mediators

Health mediators acted as facilitators between GP cluster workers and the serviced populations with the aim of increasing the access and uptake of health services among vulnerable groups (characterized by low income, primary education only, minority status or a combination of these) who have been known to be reluctant to attend such services [5, 15]. They participated in the organization and operation of various preventive services as specified by the public health coordinator (their supervisor) and the GP, being specifically responsible for increasing attendance at the health status assessment. Health mediators received a list of those who did not show up at the assessment in spite of receiving a written letter of invitation, and they had to make house visits to persuade non-attendees to participate. They carried out the majority of fieldwork related to community-based events at which they also participated, manning various posts during such events. Health mediators were also involved in various health education activities facilitated by training, relevant material and printed leaflets for distribution. Mediators also had administrative tasks of reporting their work, including participation at the monthly meetings of the GP cluster.

## Methods

Our aim was to assess the workload of health mediators and to estimate their contribution to the implementation of health status assessment and community health promoting programmes in the group practices of the Model Programme.

### Data collection and analysis

The framework of the Programme along with major indicators was specified in a bilateral agreement between the Swiss and Hungarian Governments in 2012. The percent of Roma patients accessing services had been specified as one of the major indicators. Ethnic identity was reported by patients themselves by responding to two questions taken from the 2011 Census [20] in the self-filled questionnaire of the health status assessment (HSA). The questionnaire of the HSA used items and scales from the Hungarian version of the European Health Interview Survey 2009, Hungarostudy 2013, and other validated instruments in Hungarian [8, 21–23]. The detailed development of the Model Programme (including all indicators) was carried out by a Consortium of experts delegated by nine bodies including four national health institutions and five leading universities. The programme was managed by an expert team affiliated to the national institution responsible for health care management. There had been institutional and personnel changes in the management of the Programme during 4 years of implementation (2013–2017), therefore the names of the institutes are not reported. All administrative data, including work hours and activities of all workers of the Programme, as well as monitoring of attendance of all individual, group and community services by clients was mandatorily reported on a monthly basis according to the Programme Implementation Manual (398 pages and 22 Annexes) written by the Management and approved by the Consortium. Health mediators logged their work hours and activities daily on standard forms and reported them monthly to the public health coordinator as prescribed in the Manual (along with all other employees of the GP clusters). Heads of the GP clusters were responsible for data collection and reporting in their clusters. All data from the GP clusters were sent to Management at headquarters where data processing was carried out. Data analysed in this paper were provided by Management in aggregated electronic format so as to prevent individual identification of any worker in any GP cluster.

### Work performance of health mediators

Altogether 48 part-time positions for health mediators (12 per GP cluster) had been planned during the implementation phase. However, these entry-level half-time positions provided quite low wages that led to many of the mediators leaving their jobs if they found full-time employment elsewhere. Remaining mediators repeatedly requested their work hours to be increased to that of full-time and some GP clusters did request changing the part-time contracts to that of full time (granted by the Management). In order to make the workloads among GP clusters directly comparable and to account for changes in contracts and length of employment, work time for all health mediators was calculated in work hours based on the number of positions and duration of employment between July 2013 and January 2017. Potential work hours were calculated by the number of available part-time positions; actual work hours were calculated based on the number of filled positions and by number of work hours per week per person taking into account the type of contract (part-time or full-time).

### Data on new services of GP clusters

All data on service uptake and attendance of programmes used in the analysis were made available by the Management of the Programme that was responsible for data collection throughout the Programme. Descriptive statistics and Pearson correlations were calculated in MS Excel 2016.

## Results

### Job fulfillment/vacancy

**Fig. 2:**
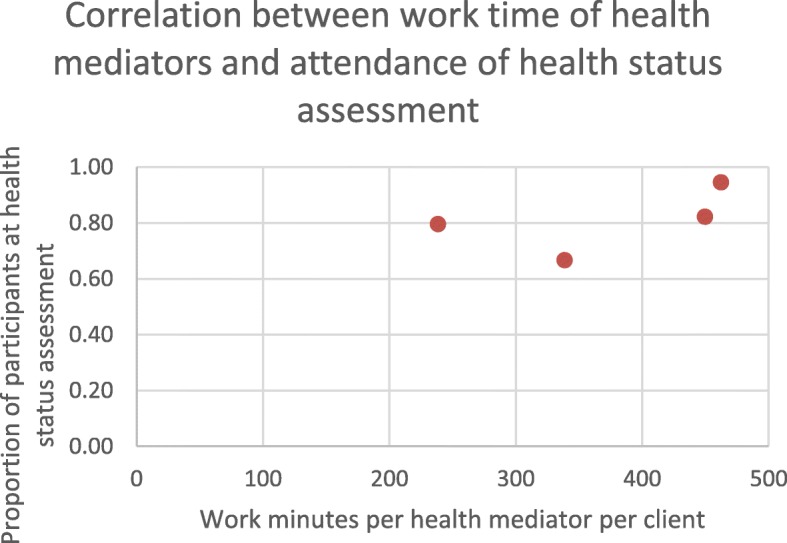
Correlation between work time of health mediators and attendance of health status assessment

As Table 1 reveals, health mediators spent most of their time recruiting for and helping with health status assessment. This activity was exclusively organized and carried out by non-medical personnel of the clusters, similar to community health promoting events, hence the non-involvement of doctors. Regarding health status assessment, health mediators had to visit and individually engage with patients who did not show up at the health status assessment after receiving written invitation. Therefore, work minutes per health mediator per patient in each cluster was calculated as independent variable to account for the different numbers of patients in the clusters for Fig. 2.

**Table 1:** Work characteristics of health mediators compared to GPs and public health specialists in the Model Programme

Work characteristics during 43 months of the Programme	GP	Public health specialist	Health mediator
Mean duration of employment (months)	36	42	32
Mean of work minutes per client considering all clients listed with the group practice (minutes)	189	56	372
Mean of work minutes per client attending health status assessment (minutes)	–	70	460
Mean of work minutes per participant attending community health promoting events (minutes)*	–	8	53
Proportion of actual work hours compared to the maximum work hours (per cent)	90	97	100
Proportion of those who left the Programme (per cent)	8	0	45

*between October 2014 – September 2015

80% of clients listed with and invited by the GPs in the Programme did attend the health status assessment. This service is provided by other GPs in Hungary only by the patient’s request, so attendance of this service could only be compared to participation at other, invitation-based national screening programmes of the country. Attendance of the health status assessment in the Model Programme was 1.3–1.7 times higher than that of national screening programmes. A correlation analysis between the number of mediator work minutes per client and the participation rate at health status assessment by GP clusters showed a strong positive linear correlation (r = 0.549) that was not significant due to the low number of datapoints (Fig. 2).

**Fig. 3:**
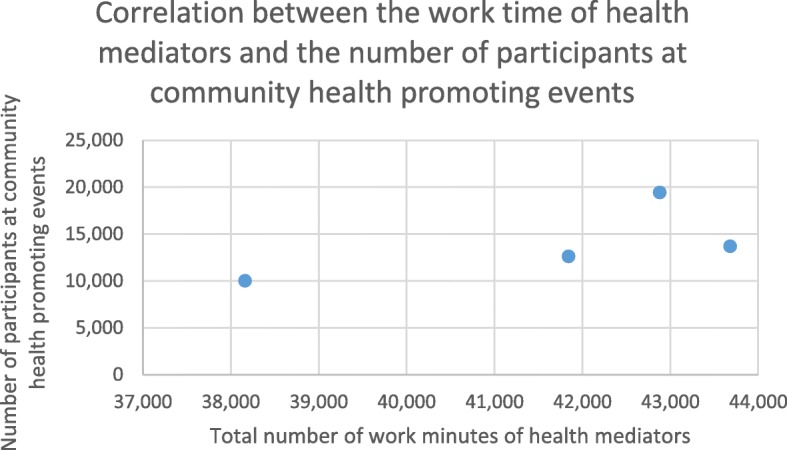
Correlation between the work time of health mediators and the number of participants at community health promoting events

Part of the mediators’ work hours was dedicated to organizing community health promoting events (data on these events are restricted to one year between October 2014 and September 2015), not requiring individual engagement with patients, but rather, organizational and logistic activities. Since this work was not dependent on the number of patients in the GP clusters, the total number of work minutes of health mediators was calculated as independent variable for Fig. 3. These events were altogether attended by 74% of all persons listed with the 4 GP clusters (Fig. 3). Pearson’s correlation coefficient was high (r = 0.713) though not significant due to the low number of datapoints for the positive association between the total number of health mediator work hours and the total number of participants at community health promoting events in the examined period in the four GP clusters.

Though the number of datapoints (each representing one GP cluster) are insufficient to support significance, the relationship of work performance (work minutes) of health mediators to the number of participants can be seen both in terms of the health status assessment (Fig. 2.) and health promoting events (Fig. 3).

As part of the programme evaluation, a patient attitude survey in a representative sample of 1022 persons was conducted in 2016 of whom 83.6% had attended health assessment. 20% of all, and 40% of Roma respondents of this survey mentioned that they attended health assessment on the recommendation of health mediators.

As new additions of the primary care workforce, health mediators had to find their niche in the group practices that took time and effort as reflected by the relatively high proportion of mediators who left the Programme during its examined 43 months (45%, Table 1). However, the proportion of job leavers was down to 20% between January 2016 and January 2017 reflecting increased integration.

Forty-eight health mediators in half-time employment entered the Programme at its start. The turnover was quite high; several new mediators were hired during 4 years. The employment status of health mediators was changed to that of full-time position if requested by the Head GPs of the clusters from the Management. By January 2017, only 66% of the mediator positions were filled (32 out of 48) but 38% of the mediators had worked full-time, reflecting their useful contribution to the services of the GP clusters.

## Discussion

Our paper gives account of the contribution of health mediators as members of multidisciplinary primary health care teams that were created in Hungary in the framework of the so-called Primary Care Model Programme from 2013. The Model Programme was the first in the country to create multidisciplinary teams in primary care, necessitated by a number of problems related to single-handed practices such as lack of preventive services, inequalities in access to primary care services, and uneven distribution and shortage of general practitioners, among others [9]. Their work was evaluated in relation to two new services that are not provided by single-handed GP practices in the country. Health assessment is carried out by GPs only if it is requested by the patient, so attendance of this service in the Model Programme can be compared to participation at other, invitation-based national screening programmes in the country. Breast cancer screening has been carried out by invitation among 45–65 year-old women every 2 years; its national rate of attendance ranged from 45% in 2015 [24] to 61% in 2002–2003 [25]. Cervical screening is requested from gynecologists by 50 to 60% of women aged 25 to 65 years. A national programme of cervical screening inviting women to attend since 2003 did not significantly increase the proportion of those who had been screened by 2010 [26]. Attendance of the health status assessment in the Model Programme was 1.3–1.7 times higher than that of these national screening programmes. Correlation analysis between the relevant indicators of mediator work time and participation at health status assessment and community health promoting events by GP clusters showed positive correlations, reflecting the substantial contribution of health mediators in the uptake of these services.

### Strengths and limitations

Indicators of traditional and new services of GP clusters had been collected during a relatively long time-period (43 months). However, separating the contribution of health mediators from other workers of the GP clusters is limited by the fact that they participated in services in which other workers had also been involved so their contribution can only be approximated. The quantification of the share of workers in primary care outcomes is possible only for those services that are provided by particular workers independently [27]. Access to the new services by Roma patients was definitively improved due to the tenacity and persistence of health mediators, but the proportion of Roma patients is based on self-identification during service uptake but not in the database of GPs since the latter is not allowed in the country.

Since the proportion of Roma in the patient attitude survey was almost three times higher than the proportion of Roma in health assessment (20% vs 7.2%), selection bias cannot be excluded, and probably resulted in a slight overestimation of the impact of health mediators on participation. However, this does not call into question the substantial motivational effect of health mediators on the participation of ethnic minority patients at health assessment.

### Health mediators as team members in primary care

The institutionalization and professionalization of health mediation had been recommended on the basis of the accumulated experiences during the Decade of Roma Inclusion [17]. The Hungarian model programme described above is the first in which these recommendations were fully implemented. Health mediators were recruited from the serviced communities, received vocational training, and became employees and equivalent members of GP group practices who facilitated the access to and uptake of services among Roma minority groups, though at the expense of putting in high numbers of work hours the sustainability of which remains to be seen. Moreover, as the unprecedentedly high participation rate in the health status assessment allows us to surmise, health mediators likely facilitated access to primary care services for many members of the community, not only to those with Roma identity. In this respect, health mediators shifted towards the role that community health workers fulfill in primary care in many countries outside of Europe [13, 14, 28]. It is of interest to note that though the WHO policy guideline on community health worker programmes was published in 2018, the system support of health mediators in the Hungarian Model Programme established in 2013 partially or fully met all 15 recommendations of the WHO guideline reflecting conceptual overlap between the two types of nonprofessional workers.

The composition and changes of the health mediator workforce will be described in a separate paper.

The Programme had planned to employ altogether 48 persons in 4 GP clusters in half-time jobs.

They were able to bridge the gap between general practitioners and their vulnerable clients attested by data on attendance of various primary care services. Additionally, twice as many Roma patients mentioned health mediators as the motivators for accessing services, underlining the importance of attending to vulnerable persons [19]. The type of work carried out by health mediators required personal contact with patients in their homes so a considerable part of the work hours of health mediators was spent on travelling on foot since most of the GPs worked in villages with no mass transportation. (Bikes were provided for mediators only in the last year of the Programme.) Based on experiences of this Programme, full-time (instead of part time) employment of health mediators can be justified along with the provision of some means of transportation in primary care to which sizable disadvantaged groups belong.

### The future of primary health care

There has been a continued debate in the past decades whether single-handed practices can remain long-term alternatives to group practices since the latter have lower structural costs, more ancillary staff, tend to provide a wider range of services, and their GPs are much less at risk of becoming professionally isolated – all pointing to higher quality of care [29]. There have been conflicting accounts whether single-handed practices provide lower quality of care compared to group practices [30, 31], but relevant reports agree that single-handed practices tend to operate in deprived areas serving clients with higher needs [32]. The special needs of populations in low socioconomic strata were also highlighted in Hungary where the rate of non-performed preventive services was found to be highest among those with no more than primary education [6].

The future of general practice as spelled out by the World Health Organization [33] and the Royal College of General Practitioners [34] lies in multidisciplinary teams that provide integrated, comprehensive, cost-effective and patient-centered care in a way that also contributes to the reduction of health inequalities at the community level. These multidisciplinary teams must expand their workforce in order to provide a wide range of services to the local communities that are easily accessed and taken up by clients from all strata of society. Health mediators – expanding their work from minority persons to that of all vulnerable patients of group practices – can not only ease the workload of GPs, a key issue of primary care [35], but can also increase patient satisfaction by bridging the physical and societal distance between health professionals and their disadvantaged patients.

## Conclusions

Health mediators as facilitators of the access and uptake of primary health services among Roma minority groups had been employed as support workers of primary care practices in a Model Programme that established group practices (GP clusters) in Hungary in 2013. The contribution of health mediators during 43 months of the Programme was reflected by the high participation rate (80%) of all clients registered with GPs at health status assessment that exceeded participation rates of other national screening programmes; as well as by the correlation of mediator work time and participation rates at health status assessments and at community health events. Health mediators recruited from the serviced communities can be valuable members of multidisciplinary primary healthcare teams, especially in deprived areas.

## Data Availability

The datasets generated and/or analysed during the current study are not publicly available due to containing personal and special data the availability and use of which is regulated by law (*2011. évi CXII. törvény az információs önrendelkezési jogról és az információszabadságról*). Data collected in the framework of the Programme is stored and managed by the National Public Health Institute of Hungary and available on reasonable request from the director of the Institute.
